# Post-marketing safety concerns with trofinetide: a disproportionality analysis of the first therapeutic agent for Rett syndrome based on the FDA adverse event reporting system (FAERS)

**DOI:** 10.3389/fphar.2026.1643906

**Published:** 2026-01-14

**Authors:** Xihui Yu, Jiahong Zhong, Zhuomiao Lin, Hongbo Fu, Yaofeng Zhang

**Affiliations:** 1 Department of Pharmacy, The Second Affiliated Hospital of Shantou University Medical College, Shantou, Guangdong, China; 2 Department of Clinical Pharmacy, Meizhou People’s Hospital (Huangtang Hospital), Meizhou, Guangdong, China

**Keywords:** autism spectrum disorder, DAYBUE, FAERS, Rett syndrome, trofinetide

## Abstract

**Objective:**

Rett syndrome (RTT) is a severe, rare and chronic disease that necessitates long-term treatment. In March 2023, trofinetide was approved by the US Food and Drug Administration as the first treatment for RTT. Because of the constraints of clinical trials, certain delayed and infrequent adverse events (AEs) may go unreported, especially in orphan disease. Further research is required to investigate the potential AE signals of trofinetide in real-world scenarios, identify rare and severe AEs associated with this treatment, and promote the safe use of trofinetide among RTT patients.

**Methods:**

The data were extracted from the FAERS database from the first quarter of 2023 to the fourth quarter of 2024. Signal mining was conducted using frequency and Bayesian methods to identify positive signals associated with trofinetide. In order to obtain similar reports, the generic name “trofinetide” and commercial name “DAYBUE” were utilized.

**Results:**

A total of 3,293,302 AE reports were collected, with 15,266 AE reports from 2,824 patients related to trofinetide designated as the primary suspected drug. All the reports were from the United States. Signal mining identified 25 system organ classes (SOCs), involving 155 preferred terms (PTs). Gastrointestinal disorders had the highest report count, with diarrhoea being the most prominent AE with high report numbers and signal strength. Several noteworthy AEs except for gastrointestinal disorders were identified which are not included on the drug label, such as nasopharyngitis, decreased appetite, weight decreased, gastroenteritis viral, influenza and irritability. Time-to-onset analysis shows that most AEs occurred in 0–30 days.

**Conclusion:**

This study unveils certain potential risks associated with trofinetide in real-world applications. Medical staffs should pay more attention to AEs of patients on the first month.

## Key points

- Key findings include significant safety signals for gastrointestinal disorders along with novel signals such as nasopharyngitis, decreased appetite, weight decreased, gastroenteritis viral, influenza and irritability.
- Paediatric patient in late motor deterioration stage (>10 and ≤18 years) had an obvious stronger signal in diarrhoea and decreased appetite after receiving treatment from trofinetide.
- Our study emphasizes the need for heightened clinical awareness of trofinetide’s potential risks and highlights the importance of personalized treatment strategies to optimize safety and efficacy.

## 1 Introduction

Rett syndrome (RTT) is a severe, rare, and early-onset neurodevelopmental disorder characterized by neurological regression and autism spectrum features (Pejhan and Rastegar, 2021; Vilvarajan et al., 2023). Because the typical symptoms in the early stages of RTT resemble those of general autism spectrum disorder (ASD), it was a subtype of ASD prior to 2013. In ASD, neuroimaging has been used extensively to assess the brain structure, connectivity, and function (Adamek et al., 2020; Luo et al., 2024). Brain magnetic resonance imaging (MRI) studies in RTT have revealed global brain atrophy and region-specific reductions in gray matter (GM)/white matter (WM), particularly in the frontal and temporal lobe, hippocampus, caudate nucleus, striatum, thalamus, midbrain, and WM tracts (Kong et al., 2022). Affected children lose intentional hand use and communication skills, become socially withdrawn and occasionally upset, and develop stereotypic hand movements (Hryniewiecka-Jaworska et al., 2025). RTT primarily affects females, with an incidence of approximately 1 in 10,000. Over 95% of patients harbor *de novo* mutations in the Methyl-CpG-Binding Protein 2 (MECP2) gene (Vilvarajan et al., 2023). Patients with RTT typically exhibit normal psychomotor development and head circumference until around 5 months of age. Subsequently, they develop language development, psychomotor retardation, stereotyped motor deficits and behaviors, and loss of social engagement before the age of 4 years (Shetty et al., 2000). Breathing dysregulation is a hallmark of RTT, affecting up to 93% of patients. This symptom significantly impairs quality of life and may contribute to early mortality (Ramirez et al., 2013). Low weight, frequent seizures, and impaired ambulation have been identified as risk factors for mortality, and the prognosis for patients with RTT has historically been dismal (May et al., 2024). Although survival rates reach 77.6% at age 20% and 59.8% at age 37 (Anderson et al., 2014), RTT is challenging to treat due to its syndromic complexity, multi-system involvement, and limited therapeutic options (Camillo et al., 2024). Annual healthcare costs exceed $40,000 for female RTT patients, with pediatric cases averaging over $45,000 (May et al., 2023).

In March 2023, trofinetide was approved by the US Food and Drug Administration (FDA) as the initial treatment for RTT in adults and pediatric patients aged ≥2 years (Percy et al., 2024b). The mechanism is presumed to involve enhancement of synaptic function and morphology, as demonstrated in MECP2 mouse models of RTT (Tropea et al., 2009). A phase 2, multicenter, double-blind, placebo-controlled, parallel-group study showed that all dose levels of trofinetide were generally safe and well-tolerated in individuals with RTT aged 5–15 years (Glaze et al., 2019). A randomized phase 3 study in a large cohort of girls and women aged 5 to 20 with RTT found that trofinetide outperformed placebo for both the coprimary and key secondary efficacy endpoints (Neul et al., 2023). Pooled safety data indicate that the most common adverse events (AEs) associated with trofinetide were diarrhea, COVID-19, and vomiting (Percy et al., 2024a; Percy et al., 2024b). Gastrointestinal AEs, in particular, may compromise adherence and negatively affect health-related quality of life. Long-term safety data are therefore essential, because RTT requires lifelong therapy (Fu et al., 2020). Post-marketing pharmacovigilance and real-world evidence studies are needed to detect rare or serious AEs, refine risk-benefit estimates, and optimize safe prescribing in the RTT population.

Because of the constraints of clinical trials, certain delayed and infrequent adverse events (AEs) may go unreported, especially in orphan diseases. The FDA Adverse Event Reporting System (FAERS) offers an open-access repository of post-marketing AEs and medication errors that is widely used for pharmacovigilance. By collating voluntary reports from health-care professionals, patients, and manufacturers worldwide, FAERS enables comprehensive real-world safety assessments (Zhong et al., 2024). We performed a commonly disproportionality analysis of FAERS data to detect safety signals associated with trofinetide and to characterize affected patients. The findings are intended to inform regulatory action, guide prescribers, and ultimately enhance the safe use of trofinetide in routine clinical practice.

## 2 Methods

### 2.1 Data source and collection

We performed a retrospective pharmacovigilance analysis on AEs of trofinetide using the FAERS database, a publicly accessible database of safety reports filed by pharmaceutical firms, pharmacists, and consumers worldwide since 2004. It is the largest spontaneous reporting system database in the world, containing more than 9 million individual drug-related adverse event reports that have been submitted by consumers, healthcare professionals, doctors, pharmacists, and industry professionals (Liu et al., 2024). The FDA launched trofinetide in March 2023, and AEs were gathered from the first quarter of 2023 to the fourth quarter of 2024. Since the FDA updates its quarterly data every 3 months and the data cleaning process takes some time to ensure its accuracy, our research has only been updated up to the fourth quarter of 2024.

### 2.2 Data processing

The FAERS data were acquired from the Quarterly Data Extract Files, which are publicly accessible at https://fis.fda.gov/extensions/FPD-QDE-FAERS/FPD-QDE-FAERS.html. We obtain the FAERS data and clean the data via RStudio following the instructions from the FDA. We searched the whole drug nomenclature of trofinetide, including trade names, generic names, non-proprietary names, and medicine brands. To find similar reports, the generic name “trofinetide” and commercial name “DAYBUE” were utilized. The following brand or generic names of the medications were filtered out of the database by the study: “trofinetide”, “DAYBUE” and “NNZ-2566”. Only the reports of trofinetide with role code as the primary suspected drug were chosen for analysis. When referring to the names of AEs in the reports, preferred terms (PTs) from the Medical Dictionary for Regulatory Activities (MedDRA) should be used for consistent encoding. The study included all PTs that fell within the larger category of diseases and infestations known as system-organ classes (SOC). Because FAERS data often contain duplicates, we keep the report with the highest FDA_DT value for reports with the same CASEID. We keep the report with the highest PRIMARYID value when the CASEID and FDA_DT are same. FDA_DT refers to the date when the FDA received the report. CASEID refers to different individuals while PRIMARYID represents the report number. It should be noted that an individual may have multiple reports of AE, so an CASEID could have different PRIMARYID. The larger value of PRIMARYID means that the reported date is more recent. A final dataset that is prepared for analysis was created by compiling the cleaned and standardized data. Supplementary Figure S1 depicts the comprehensive screening procedure. In keeping with the emphasis of our analysis, this dataset only contained cases in which trofinetide was identified as the primary suspected drug (PS). Every AE report of trofinetide was examined at the System Organ Class (SOC) and PT levels.

### 2.3 Statistical analysis

Disproportionality analysis is a useful tool for identifying and detecting drug-related adverse reaction signals in pharmacovigilance studies (van Puijenbroek et al., 2002), and methods with high sensitivity can identify more potential AEs and reduce the likelihood of missing true signals, while methods with high specificity can reduce the proportion of false positive signals (Jiao et al., 2024). To increase the reliability of the results, we used various disproportionality analysis techniques to reduce the bias of false-positive results caused by one method, such as reporting odds ratio (ROR) and proportional reporting ratio (PRR), Bayesian confidence propagation neural network (BCPNN), and multi-item gamma Poisson shrinker (MGPS). ROR and PRR methods with high sensitivity were selected in order to mine more ignored adverse reaction signals in this paper. At the same time, in order to avoid the misleading of false positive signals, we chose BCPNN and MGPS methods with high specificity to ensure the robustness of the results. A preferred terminology is considered a positive signal if it simultaneously meets the thresholds of all four algorithms, and the equations and criteria for the four algorithms are detailed in Supplementary Table S1. Data were analyzed using Microsoft Excel 2021 and R 4.3.0. We conducted a sensitivity analysis and controlled for confounding factors by performing subgroup analyses based on age.

### 2.4 Time to onset analysis

The gap between EVENT_DT (the date of AE onset in the DEMO file) and START_DT (the date of medication commencement in the THER file) was used to calculate the time to onset (TTO) of trofinetide-related AEs. Excluded were cases with mistakes (not specific to a day, month, or year) or missing dates (either the start of AEs or the start of treatment). Furthermore, because this would provide a negative time-to-onset computation, instances where the starting date of AEs occurred prior to the start date of trofinetide medication were also eliminated (Liu et al., 2024). In this work, we measured TTO features using the median, quartile, minimum, maximum, and Weibull shape parameter (Kinoshita et al., 2020).

## 3 Results

### 3.1 General characteristics

From the first quarter of 2023 to the fourth quarter of 2024, the FAERS database received a total of 3,293,302 reports. Following data deduplication and screening, 15,266 AE reports involving 2,824 patients were identified, with trofinetide designated as the PS drug.

Clinical characteristics of AEs to trofinetide are shown in Table 1. In terms of gender, approximately 92.9% patients were female and 3.4% were male. In addition to the majority of unknown ages, most reports focused on the age group below 10 years (17.70%). In terms of reporting sources, most reports were provided by consumer (96.9%). AEs were reported primarily in the United States. Excluding unknown outcome, other serious (important medical event) get the most reports (16.5%). Since its launch in 2023, the number of reported AEs has shown a steady annual increase, peaking in 2024 with 88.8% of the total reports.

**TABLE 1:** Characteristics of reports associated with trofinetide from the FAERS database (Q1 2023-Q4 2024).

Factors	Number of patients (%)
Case reports	2,824 patients with 15,266 AE reports
Gender	
Female	2,624 (92.9)
Male	96 (3.4)
Unknown	104 (3.7)
Age (year)	
<2	6 (0.2)
≥2 and ≤10	494 (17.5)
>10 and ≤18	304 (10.8)
>18	350 (12.4)
Unknown	1,670 (59.1)
Reporter	
Consumer	2,737 (96.9)
Health professional	44 (1.6)
Physician	39 (1.4)
Pharmacist	4 (0.1)
Reporter country	
United States	2,824 (100)
Outcome	
Other serious (important medical event)	465 (16.5)
Hospitalization - initial or prolonged	337 (11.9)
Death	15 (0.5)
Life-threatening	5 (0.2)
Unknown	2,002 (70.9)
Reporting year	
2023	315 (11.2)
2024	2,509 (88.8)

### 3.2 Signal detection of trofinetide at the system organ class (SOC) level

**FIGURE 1:**
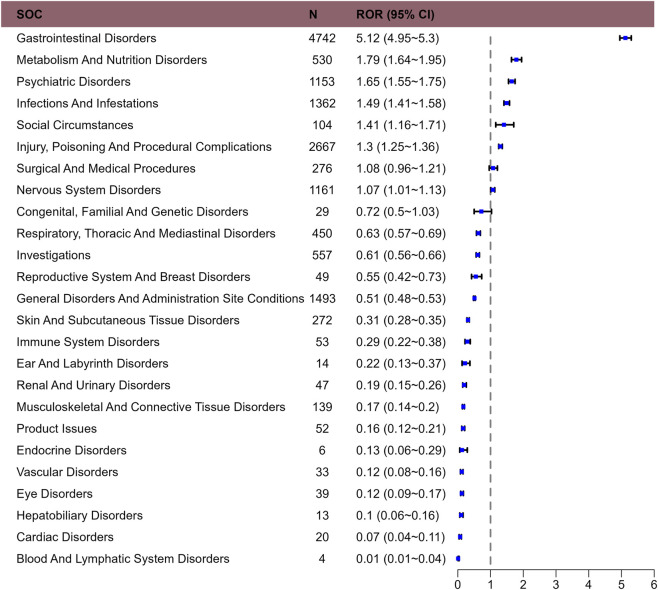
Reporting odds ratios with 95% CI for adverse events at the System Organ Class level.

The signal strength of trofinetide at the SOC level is shown at Table 2. After conducting an analysis, we have identified a total of 25 SOCs that are affected by adverse drug reactions caused by trofinetide in Figure 1. The most frequently reported SOC was gastrointestinal disorders. It demonstrated a strong positive signal in ROR, aligning with descriptions in the trofinetide drug label, which suggests high data reliability.

**TABLE 2:** Multi-method disproportionality analysis of AEs associated with trofinetide above 100 cases at the PT level with positive signals.

SOC	PT (preferred term)	N	ROR (95%Cl)	IC (IC025)	PRR (χ^2^)	EBGM (EBGM05)
Gastrointestinal disorders	Diarrhoea	1,913	13.21 (12.58–13.86)	3.52 (3.45)	11.68 (18529.16)	11.48 (11.02)
	Vomiting	629	6.37 (5.88–6.9)	2.61 (2.49)	6.15 (2703.62)	6.1 (5.7)
	Flatulence	228	19.02 (16.65–21.71)	4.19 (3.99)	18.75 (3719.09)	18.22 (16.3)
	Faeces soft	219	72.76 (63.2–83.78)	6.01 (5.8)	71.73 (13670.39)	64.29 (57.14)
	Retching	212	50.3 (43.69–57.91)	5.52 (5.32)	49.62 (9341.97)	45.96 (40.85)
	Abdominal discomfort	153	3.58 (3.05–4.2)	1.82 (1.59)	3.55 (279.65)	3.54 (3.09)
Infections and infestations	Nasopharyngitis	202	3.66 (3.18–4.2)	1.85 (1.65)	3.62 (382.31)	3.61 (3.21)
	Gastroenteritis viral	198	39.58 (34.25–45.74)	5.2 (4.99)	39.08 (6907.14)	36.79 (32.6)
	Influenza	103	3.17 (2.61–3.85)	1.65 (1.37)	3.16 (151.41)	3.15 (2.67)
Investigations	Weight decreased	299	4.45 (3.97–4.99)	2.12 (1.95)	4.38 (778.5)	4.36 (3.96)
Metabolism and nutrition disorders	Decreased appetite	217	3.69 (3.22–4.22)	1.86 (1.66)	3.65 (416.07)	3.63 (3.25)
	Hypophagia	145	26.51 (22.43–31.33)	4.66 (4.41)	26.27 (3379.8)	25.22 (21.93)
Psychiatric disorders	Irritability	124	13.86 (11.59–16.57)	3.75 (3.49)	13.75 (1434.92)	13.47 (11.6)

### 3.3 Signal detection of trofinetide at the preferred terms (PTs) level

The results showed that 155 PTs met the positive criteria across all four algorithms in Supplementary Figure S2. At the PT level, we also found that seizure, constipation, gastroesophageal reflux disease, crying, insomnia and somnolence were significant in the disproportionality analyses, which are all symptoms and comorbidities of RTT (Neul et al., 2023; Gold et al., 2024). Therefore, for the accuracy of the study results, we excluded the AEs from our results. The AEs associated with trofinetide above 100 cases at the PT level is shown at Table 2. AEs ranks the SOCs by the number of reports at Table 2, with the highest number for gastrointestinal disorders (n = 3,354), followed by infections and infestations (n = 503), metabolism and nutrition disorders (n = 362), investigations (n = 299) and psychiatric disorders (n = 124). The definition of “investigation” in MedDRA is a clinical laboratory test concept, radiologic test concept, physical examination parameter, and physiologic test concept. For example, weight decreased is in the category of “investigation”.

Among the common AEs, several events were identified that aligned with those listed on the drug label, including diarrhoea and vomiting. Additionally, several noteworthy AEs except for gastrointestinal disorders were identified which are not included on the drug label, such as nasopharyngitis, decreased appetite, weight decreased, gastroenteritis viral, influenza and irritability.

### 3.4 Age-stratified analysis of adverse events

**FIGURE 2:**
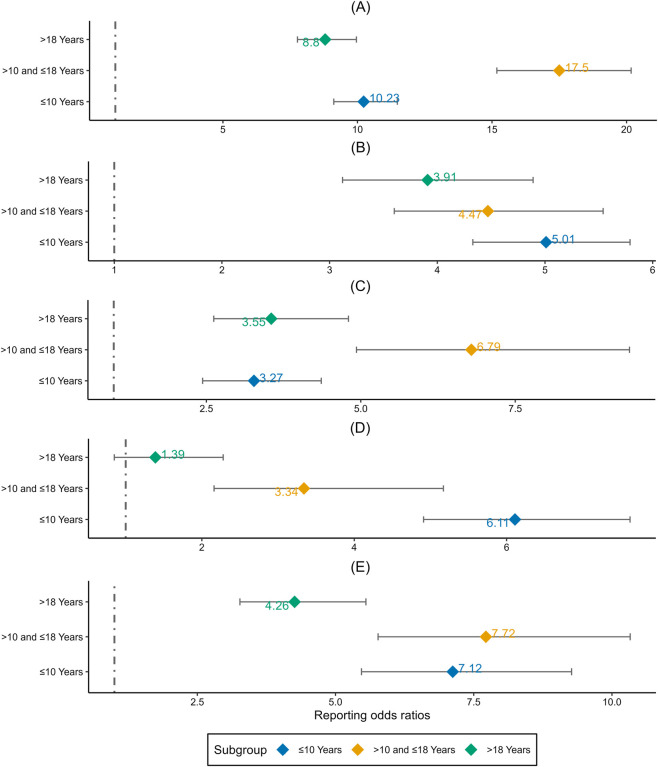
Age-stratified analysis of adverse events associated with trofinetide: a reporting odds ratio analysis in three age groups. **(A)** Diarrhoea. **(B)** Vomiting. **(C)** Decreased Appetite. **(D)** Nasopharyngitis. **(E)** Weight decreased.

The course of RTT in its classical form is characterized by four stages, which are early onset (6 months to 1 year old), rapid destructive (1 year to 3 years old), plateau (2 years to 10 years) and late motor deterioration (more than 10 years) (Operto et al., 2019). Due to the sample size in the early onset and rapid destructive stages, we have grouped the three stages of early onset and rapid destructive and plateau together (≤10 years). The remaining population of late motor deterioration was divided based on children and adults. We performed age-stratified analysis of adverse events to investigate whether there were differences according to different stages in three groups (≤10, >10–18, >18 years). In addition, we also performed subgroup analyses between children and adults. As shown in Figure 2, pediatric patient in late motor deterioration stage had a stronger signal in diarrhoea and decreased appetite.

### 3.5 Time-to-onset (TTO) analysis

**FIGURE 3:**
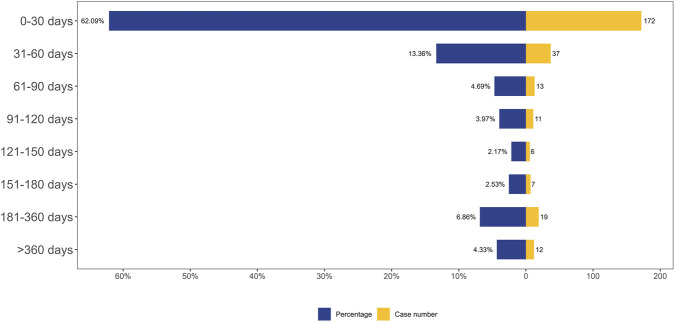
TTO analysis of trofinetide-related AEs counted in days.

A total of 277 AEs were associated with effective TTO reports. The distribution of onset times for these AEs is shown in Figure 3. The median of TTO was determined as 19 days and the interquartile range (IQR) was 4–58 days. As shown in Figure 3, most cases occurred in 0–30 days (n = 172, 62.09%) of trofinetide administration. These findings emphasize the importance of monitoring patients for potential AEs beyond the initial months.

We performed Weibull distribution tests on both the whole patient population in Supplementary Table S3 and the trofinetide-associated adverse events to see if the risk of these events rises or falls with time. An early failure-type curve is thought to indicate a decreasing likelihood of negative effects with time when the form parameter β is less than 1 and its 95% confidence interval (CI) is likewise below 1 (Mazhar et al., 2021). The Weibull distribution test for TTO indicated that the upper limit of the 95% CI for the shape parameter (β) was 0.61 (less than 1), suggesting that the probability of AEs gradually decreased over time.

**FIGURE 4:**
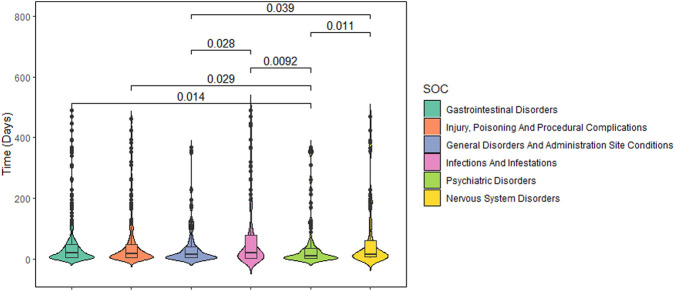
Violin plot of onset time at the top 6 of System Organ Class.

Additionally, we analyzed the TTO reports at the SOC level. SOCs with the shortest median onset times (MOT) was psychiatric disorders (MOT = 9 days), with a significant difference between gastrointestinal disorders (MOT = 19 days, *P* = 0.014), injury, poisoning and procedural complications (MOT = 17 days, *P* = 0.029), infections and infestations (MOT = 21 days, *P* = 0.009) and nervous system disorders (MOT = 15 days, *P* = 0.011), respectively (Figure 4; Supplementary Table S4).

## 4 Discussion

### 4.1 Baseline information description

Our findings show that most trofinetide-related side effects occur in females (92.9%) than in males (3.4%). This is related to the fact that the population affected by Rett syndrome is mainly female because Rett syndrome is a rare genetic X-linked neurodevelopmental disorder (Palmieri et al., 2023). Among all age groups, the higher incidence of AEs in patients below 10 years old may be related to the clinical characteristics of this population and the median age of diagnosis was 3 years old (Vilvarajan et al., 2023). Adverse drug reactions were valued by consumers (96.9%), indicating a high patient focus on drug safety and possibly reflecting high expectations for the treatment of Rett syndrome.

### 4.2 Known AEs

In this investigation, there were 155 AEs with positive signals. The present study found that the AEs associated with trofinetide were mainly centered on the gastrointestinal system, such as diarrhoea and vomiting. A long-term safety and efficacy results of the 32-month, open-label LILAC-2 study showed that diarrhea was the most frequently reported AE in participants with RTT treated with trofinetide (Percy et al., 2024b). Diarrhea was the most common AE leading to treatment discontinuation from the open-label extension LILAC study (Percy et al., 2024a). According to an FDA statement, vomiting and diarrhea are frequent side effects of the twice-daily oral medication, which is consistent with our findings (Harris, 2023). The cause of diarrhea and vomiting with trofinetide use is unknown but trofinetide may have a neuroendocrine effect in the gut, which is similar to glucagon-like peptide-1 receptor agonist drugs that may cause diarrhea or constipation (Motil et al., 2024). Furthermore, diarrhea may also be caused by the presence of maltitol in the current formulation of trofinetide. Maltitol is a sugar alcohol that is broken down in the intestines into glucose and sorbitol, thereby increasing the osmotic load in the colon (Motil et al., 2024). There are some recommendations on the management of diarrhea with the use of trofinetide. Evidence-based strategies for trofinetide-associated diarrhea include immediate initiation of weight- and age-adjusted oral loperamide (Marsh et al., 2023). If symptoms persist, clinicians should reduce the dose of trofinetide to the last tolerated dose. In the event of vomiting with trofinetide treatment, management approaches include reducing the volume of liquids before and after trofinetide administration and reducing or dividing the dose of trofinetide (Motil et al., 2024).

### 4.3 New potential AEs

The study identified previously unlisted infections symptoms during trofinetide treatment, including nasopharyngitis, gastroenteritis viral and influenza. While the mechanism of action of trofinetide has yet to be fully elucidated, due to its homology with the N-terminus of Insulin-Like Growth Factor-1 (IGF-1), it is hypothesized that it acts through the IGF-1 receptor (IGF-1R) (Parent et al., 2023). IGF-1 binding to the IGF-1R is involved in a mechanism of respiratory syncytial virus (RSV) entry into cells involving respiratory syncytial virus fusion (RSV-F) glycoprotein expressed on the virion surface (Griffiths et al., 2020), which may be the mechanism of influenza inducing by trofinetide.

Decreased appetite and weight decreased could be caused by diarrhoea and vomiting (Camillo et al., 2024). 12% of subjects on long-term treatment with trofinetide experienced a loss of greater than 7% of body weight (Camillo et al., 2024). A study in chicks suggests that hypothalamic proopiomelanocortin (POMC) and v-Akt murine thymoma viral oncogene homolog 1 (AKT1) may be involved in the IGF-1-induced anorexigenic pathway (Fujita et al., 2019). IGF-1 and proinsulin have structural similarities. IGF-1 can not only cause a decrease in blood sugar levels, but also reduce insulin levels (Laron and Werner, 2025). Decrease in insulin levels might be related to the fact that trofinetide caused a decrease in weight.

Psychiatric disorders showed rare but strong signals after receiving treatment of trofinetide. A systematic review and meta-analysis of randomized controlled trials showed that a statistically significant increase in irritability occurrence in the trofinetide group (Abbas et al., 2024), which is consistent with our findings. The specific mechanism of irritability in RTT patients treated with trofinetide has not been reported in the literature, and further research is needed. The reduction in insulin influenced by IGF-1 may have an impact on the regulation of emotions (Mueller et al., 2018).

### 4.4 Age-based difference in risk signals for trofinetide

Pediatric patient in late motor deterioration stage (>10 and ≤18 years) had an obvious stronger signal in diarrhoea and decreased appetite. This finding reflects that there may be some correlation between diarrhoea and decreased appetite. The main symptoms during late motor deterioration stage are reduced mobility, muscle weakness and stiffness, stiffness in joints, scoliosis (abnormal curvature of the spine), and loss of walking ability (Camillo et al., 2024). It is suggested that doctors should pay close attention to the diarrhea of patients in late motor deterioration stage after using the drug, and adjust the dosage of trofinetide in time.

### 4.5 TTO analysis

Since it identifies certain risk windows and promotes the avoidance or early identification of adverse responses, the temporal link between administration and time of beginning is essential for evaluating medication safety. According to TTO study, the median time of trofinetide-related AEs was 19 days, and the majority of instances (n = 172, 62.09%) happened after 1 month of trofinetide medication. The findings underscore the necessity of close adverse-event monitoring at treatment initiation.

Psychiatric disorders had the shortest MOT (9 days), which indicates an earlier occurrence of irritability. This suggests that physicians should pay attention to psychiatric disorders of patients first in the early stage of medication of trofinetide. Except for psychiatric disorders, there was no significant difference between most of other AEs, with a MOT about 20 days.

## 5 Limitations

Although many reports were gathered from the FAERS database for this study to assess the AEs of trofinetide from a variety of angles, there are still certain restrictions. Firstly, the data in the FAERS database predominantly originate from American populations (100%), with comparatively few reported data from other groups. It should be noted that different populations in different countries may have different sensitivity to trofinetide, resulting in different AEs effect. Secondly, the data in the FAERS database are spontaneously provided mainly by consumers (96.9%), with different data quality, correctness, and completeness. Reporting bias, underreporting, and incomplete data can result in an overrepresentation of certain adverse events while underestimating others. Thirdly, FAERS is not suitable for stratified analyses by RTT disease stage because of the scarcity of cases in some stages. Fourthly, time-to-onset (TTO) analysis at the SOC level is extremely underpowered because there was no significant difference between most of other AEs except for psychiatric disorders. Fifthly, some of the recorded data on serious adverse events can only be viewed from outcome in Table 1, while the outcomes were unknown in 70.9% of the cases. The incomplete data reported is an inevitable pain point in database analysis, and this is related to the mode of voluntary reporting. Furthermore, the reports of AEs that occurred later were relatively less. This might also be related to patients’ adaptation to AEs or the decline in patients’ compliance caused by diarrhoea. These would affect our ability to accurately capture new adverse effects. Finally, newer analysis methods had not yet been incorporated such as shrinkage-based empirical Bayes approaches or data-mining algorithms incorporating machine learning. Because FAERS-based safety signal analyses cannot establish causality or incidence, the discussion on the mechanism of adverse events only involves potential possibilities. These factors necessitate cautious interpretation of our findings and underscore the need for continuous and multifaceted pharmacovigilance efforts.

## 6 Conclusion

As the first drug approved specifically for the treatment of patients with RTT, trofinetide offers new hope for the rare genetic neurodevelopmental disorder. This study highlights certain safety risks associated with trofinetide in clinical applications, providing a solid scientific foundation for the safety assessment of trofinetide. Gastrointestinal disorders are the most known AEs. Newly identified potential infections (e.g., nasopharyngitis, gastroenteritis viral, influenza) suggest that trofinetide have profound effects on the respiratory system and gastrointestinal system in certain patients. To ensure the safe application of trofinetide, enhanced monitoring of AEs in high-risk populations is essential (e.g., pediatric patient in late motor deterioration stage had the susceptibility of diarrhoea and decreased appetite after the trofinetide). Medical staffs should pay more attention to AEs in the first month. If a serious adverse event occurs, the doctor should weigh the pros and cons and promptly reduce the patient’s medication dosage.

## Data Availability

Publicly available datasets were analyzed in this study. This data can be found here: https://fis.fda.gov/extensions/FPD-QDE-FAERS/FPD-QDE-FAERS.html.
