# Supplementary material for: Post-marketing safety concerns with trofinetide: a disproportionality analysis of the first therapeutic agent for Rett syndrome based on the FDA adverse event reporting system (FAERS)
Source: Front Pharmacol. 2026 Jan 14;17:1643906. doi: 10.3389/fphar.2026.1643906 (PMC12847357; doi:10.3389/fphar.2026.1643906)
Supplement: Supplementary file 1 [file Supplementaryfile1.docx]

**1 Supplementary Figures**


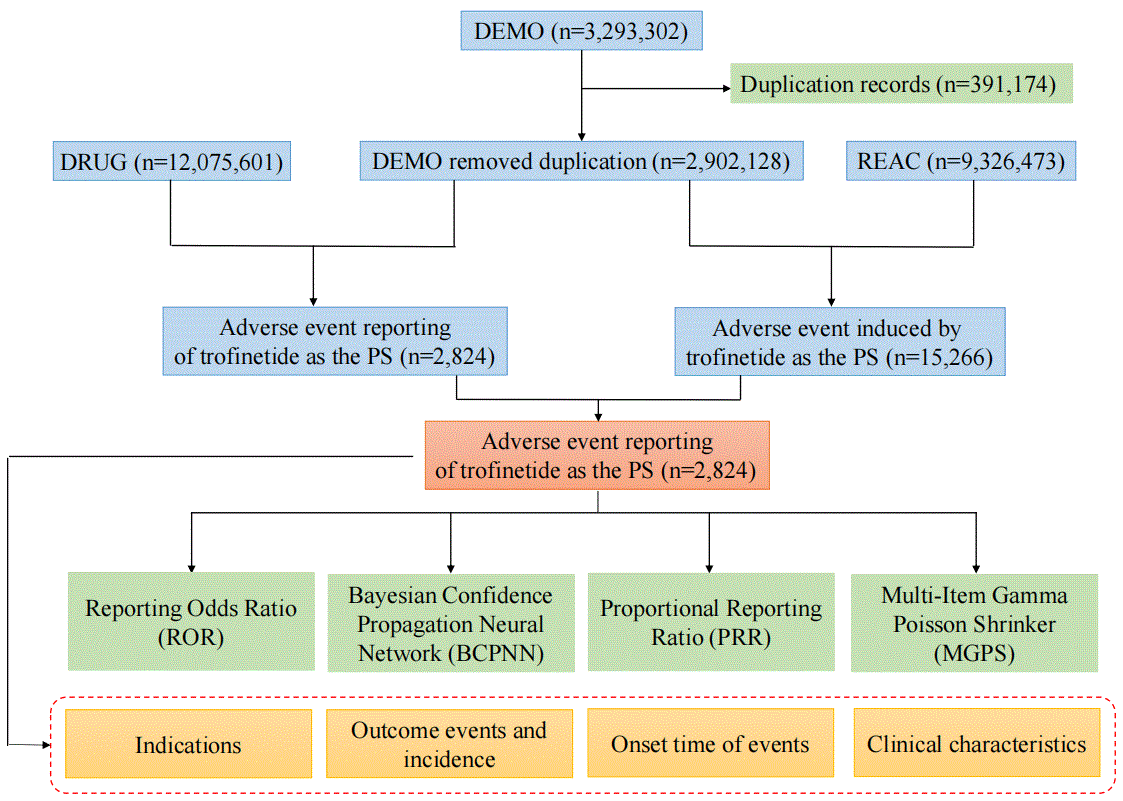


**Supplementary Figure 1**. Flow diagram of the study (DEMO, demographic and administrative information; DRUG, drug Information; REAC, preferred terminology for adverse drug reactions; PS, primary suspect drug).


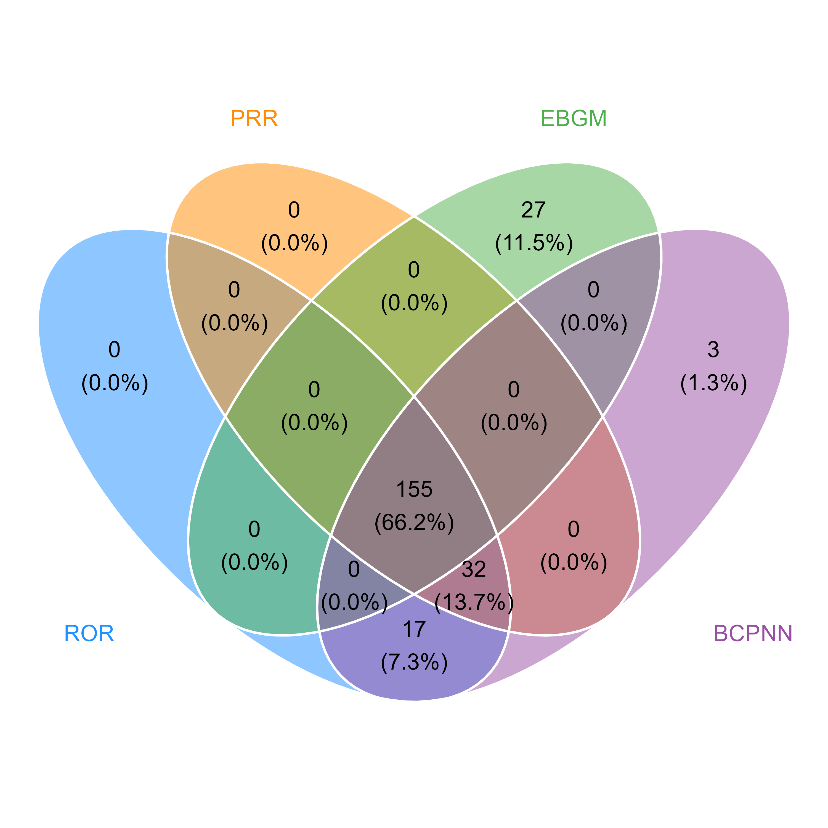


**Supplementary Figure 2**. Venn diagram of preferred term (PT) signals meeting the criteria of four algorithms.

**2 Supplementary Tables**

**Supplementary Table 1**. Calculation Formula and Standard of Signal Detection

| Algorithm | Calculation Formula | Criterion |
| --- | --- | --- |
| ROR | $\mathrm{ROR}=\frac{a/c}{b/d}=\frac{ad}{bc}$ | a ≥ 3 |
|  | $95\%CI=e^{ln(ROR)\pm1.96\sqrt{\frac{1}{a}+\frac{1}{b}+\frac{1}{c}+\frac{1}{d}}}$ | 95%CI (lower limit) > 1 |
| PRR | $\mathrm{PRR}=\frac{a/(a+b)}{c/(c+d)}$ | a ≥ 3, PRR ≥ 2 |
|  | $\chi^{2}=\frac{\left( ad-bc \right)^{2}(a+b+c+d)}{(a+b)(a+c)(c+d)(b+d)}$ | χ^2^ ≥ 4 |
| EBGM | $\mathrm{EGBM}=\frac{a(a+b+c+d)}{(a+b)(a+c)}$ | EBGM05 > 2 |
|  | $EBGM05=e^{\ln\left( EBGM \right)-1.64\sqrt{\frac{1}{a}+\frac{1}{b}+\frac{1}{c}+\frac{1}{d}}}$ |  |
| BCPNN | $\mathrm{IC}={log}_{2}\frac{a(a+b+c+d)}{(a+b)(a+c)}$ | a ≥ 3 |
|  | $E\left( IC \right)={log}_{2}\frac{(a+\gamma_{11})(N+\alpha)(N+\beta)}{(N+\gamma)(a+b+\alpha_{1})(a+c+\beta_{1})}$  $V\left( IC \right)=\frac{1}{{(ln2)}^{2}}[\frac{N-a+\gamma-\gamma_{11}}{(a+\gamma_{11})(N+1+\gamma)}+\frac{N-a-b+\alpha-\alpha_{1}}{(a+b+\alpha_{1})(N+1+\alpha)}+\frac{N-a-c+\beta-\beta_{1}}{(a+c+\beta_{1})(N+1+\beta)}]$  $\gamma=\gamma_{11}\frac{(N+\alpha)(N+\beta)}{(a+b+\alpha_{1})(a+c+\beta_{1})}$  $95\%CI=E(IC)\pm1.96\sqrt{V(IC)}$  Where α=α_1_+α_2_, β=β_1_+β_2_, N=a+b+c+d, and the value of α_1_, α_2_, β_1_, β_2_ and γ_11_ were defined as 1. | The lower limit of 95%CI (IC025) > 0 |

Abbreviations:

a, number of reports containing both the target drug and target adverse drug reaction;

b, number of reports containing other adverse drug reaction of the target drug;

c, number of reports containing the target adverse drug reaction of other drugs;

d, number of reports containing other drugs and other adverse drug reactions.

95%CI, 95% confidence interval;

χ^2^, chi-squared;

IC, information component;

IC025, the lower limit of 95% CI of the IC;

EBGM05, the lower limit of 90% CI of the EBGM.

**Supplementary Table 2**. The signal strength of trofinetide at the System Organ Class (SOC) level.

| System Organ Class (SOC) | Case number | ROR (95%Cl) | PRR (χ2) | EBGM (EBGM05) | IC (IC025) |
| --- | --- | --- | --- | --- | --- |
| Gastrointestinal disorders | 4742 | 5.12(4.95-5.3) | 3.84(10776.77) | 3.82(3.71) | 1.93(1.89) |
| General disorders and administration site conditions | 1493 | 0.51(0.48-0.53) | 0.56(645.48) | 0.56(0.53) | -0.85(-0.93) |
| Psychiatric disorders | 1153 | 1.65(1.55-1.75) | 1.6(271.94) | 1.6(1.52) | 0.68(0.59) |
| Injury, poisoning and procedural complications | 2667 | 1.3(1.25-1.36) | 1.25(156.16) | 1.25(1.21) | 0.32(0.26) |
| Infections and infestations | 1362 | 1.49(1.41-1.58) | 1.45(201.43) | 1.45(1.38) | 0.53(0.45) |
| Nervous system disorders | 1161 | 1.07(1.01-1.13) | 1.06(4.67) | 1.06(1.01) | 0.09(0) |
| Respiratory, thoracic and mediastinal disorders | 450 | 0.63(0.57-0.69) | 0.64(96.79) | 0.64(0.59) | -0.65(-0.79) |
| Surgical and medical procedures | 276 | 1.08(0.96-1.21) | 1.07(1.46) | 1.07(0.97) | 0.1(-0.07) |
| Product issues | 52 | 0.16(0.12-0.21) | 0.16(227.24) | 0.16(0.13) | -2.61(-3.01) |
| Musculoskeletal and connective tissue disorders | 139 | 0.17(0.14-0.2) | 0.17(571.47) | 0.17(0.15) | -2.52(-2.76) |
| Metabolism and nutrition disorders | 530 | 1.79(1.64-1.95) | 1.76(177.83) | 1.76(1.64) | 0.82(0.69) |
| Immune system disorders | 53 | 0.29(0.22-0.38) | 0.29(92.64) | 0.29(0.23) | -1.78(-2.17) |
| Social circumstances | 104 | 1.41(1.16-1.71) | 1.41(12.31) | 1.41(1.2) | 0.49(0.21) |
| Skin and subcutaneous tissue disorders | 272 | 0.31(0.28-0.35) | 0.33(400.5) | 0.33(0.3) | -1.62(-1.79) |
| Investigations | 557 | 0.61(0.56-0.66) | 0.62(137.63) | 0.62(0.58) | -0.69(-0.81) |
| Reproductive system and breast disorders | 49 | 0.55(0.42-0.73) | 0.55(18) | 0.55(0.44) | -0.86(-1.27) |
| Cardiac disorders | 20 | 0.07(0.04-0.11) | 0.07(251.19) | 0.07(0.05) | -3.83(-4.46) |
| Renal and urinary disorders | 47 | 0.19(0.15-0.26) | 0.2(157.65) | 0.2(0.15) | -2.35(-2.77) |
| Vascular disorders | 33 | 0.12(0.08-0.16) | 0.12(220.99) | 0.12(0.09) | -3.08(-3.57) |
| Ear and labyrinth disorders | 14 | 0.22(0.13-0.37) | 0.22(38.72) | 0.22(0.14) | -2.18(-2.92) |
| Eye disorders | 39 | 0.12(0.09-0.17) | 0.12(249.06) | 0.12(0.09) | -3.02(-3.48) |
| Blood and lymphatic system disorders | 4 | 0.01(0.01-0.04) | 0.01(268.76) | 0.01(0.01) | -6.08(-7.38) |
| Congenital, familial and genetic disorders | 29 | 0.72(0.5-1.03) | 0.72(3.24) | 0.72(0.53) | -0.48(-1.01) |
| Hepatobiliary disorders | 13 | 0.1(0.06-0.16) | 0.1(111.71) | 0.1(0.06) | -3.38(-4.15) |
| Endocrine disorders | 6 | 0.13(0.06-0.29) | 0.13(34.79) | 0.13(0.07) | -2.93(-4.02) |
| Neoplasms benign, malignant and unspecified (incl cysts and polyps) | 1 | 0(0-0.02) | 0(282.25) | 0(0) | -8.12(-10.16) |

**Supplementary Table 3**. Weibull distribution tests on TTO analysis.

| Cases |  | TTO (days) | |  | Weibull distribution | | | | |  | Failure type |
| --- | --- | --- | --- | --- | --- | --- | --- | --- | --- | --- | --- |
|  |  |  |  |  | Scale parameter | |  | Shape parameter | |  |  |
|  |  | Media (IQR) | Min-Max |  | α | 95% CI |  | β | 95% CI |  |  |
| 277 |  | 19 (4-58) | 1-489 |  | 41.17 | 32.72-49.62 |  | 0.61 | 0.55-0.66 |  | Early failure |

**Supplementary Table 4**. Interquartile range (IQR) of the TTO at the top 6 of SOC level

| SOC | Q1 | Q2 | Q3 | IQR |
| --- | --- | --- | --- | --- |
| Gastrointestinal disorders | 4 | 19 | 48 | 44 |
| Injury, poisoning and procedural complications | 4 | 17 | 47 | 43 |
| General disorders and administration site conditions | 4 | 14 | 40 | 36 |
| Infections and infestations | 3 | 21 | 77.5 | 74.5 |
| Psychiatric disorders | 2 | 9 | 36 | 34 |
| Nervous system disorders | 6 | 15 | 60.5 | 54.5 |
